# Reply to Iqhrammullah, M. Comment on “Bokayeva et al. Vitamin Status in Patients with Phenylketonuria: A Systematic Review and Meta-Analysis. *Int. J. Mol. Sci.* 2024, *25*, 5065”

**DOI:** 10.3390/ijms27052175

**Published:** 2026-02-26

**Authors:** Małgorzata Jamka, Kamila Bokayeva, Dariusz Walkowiak, Monika Duś-Żuchowska, Karl-Heinz Herzig, Jarosław Walkowiak

**Affiliations:** 1 Poznan University of Medical Sciences, Department of Pediatric Gastroenterology and Metabolic Diseases, Szpitalna Str. 27/33, 60-572 Poznań, Poland; mjamka@ump.edu.pl (M.J.); kamila.bokayeva@gmail.com (K.B.); mduszuchowska@ump.edu.pl (M.D.-Ż.); karl-heinz.herzig@oulu.fi (K.-H.H.); 2 Poznan University of Medical Sciences, Department of Organization and Management in Health Care, Marii Magdaleny Str. 14, 61-861 Poznań, Poland; dariuszwalkowiak@ump.edu.pl; 3 Research Unit of Biomedicine and Internal Medicine, Faculty of Medicine, University of Oulu, Aapistie Str. 5, 90220 Oulu, Finland; 4 Biocenter Oulu, University of Oulu, Aapistie Str. 5, 90220 Oulu, Finland; 5 Medical Research Center, Oulu University Hospital, Aapistie Str. 5, 90220 Oulu, Finland

We thank the author for their comment [1], addressing our study entitled ‘Vitamin Status in Patients with Phenylketonuria: A Systematic Review and Meta-Analysis’, published in the *International Journal of Molecular Sciences* [2]. Our meta-analysis aimed to compare vitamin status in patients with phenylketonuria (PKU) and healthy subjects. Specifically, the PKU subjects presented higher folate and 1,25-dihydroxyvitamin D (1,25(OH)_2_D) levels than the control group. However, no differences were observed between groups for the other assessed outcomes, including 25-hydroxyvitamin D (25(OH)D), a marker of vitamin D status. This suggests that individuals with PKU can attain adequate vitamin status similar to that of their healthy counterparts [2].

It is worth noting that we used the same terminology in our manuscript as the authors of the studies included in the systematic review, which may have led to the erroneous conclusion in the comment [1] that different vitamin metabolites were measured. As we did not address this issue in our manuscript [2], we would like to take this opportunity to clarify this. We decided to include in our systematic review all studies that claimed to assess any of the following vitamin D metabolites: 25-hydroxyvitamin D (25(OH)D), 25-hydroxyvitamin D_3_ (25(OH)D_3_), cholecalciferol (vitamin D_3_), 1,25-dihydroxyvitamin D (1,25(OH)_2_D) or 1,25-dihydroxyvitamin D_3_ (1,25(OH)_2_D_3_). This reflected the heterogeneity in the nomenclature used in published papers included in the meta-analysis. Despite the different terminology used across studies, most studies, except one [3], appeared to have ultimately measured 25(OH)D or 1,25(OH)_2_D. After excluding the study in which cholecalciferol concentrations were assessed, the sensitivity analysis showed no changes in the meta-analysis results. The conclusion regarding the metabolites actually measured is supported by an analysis of the reported measurement methods used in the included studies. For example, Nagasaka et al. [4,5] used identical analytical methods in both of their studies. In a 2011 study [4], the authors reported that ‘serum 1,25-hydroxyVD and 25-hydroxy VD levels were determined using RIA kits from Immunodiagnostic Systems Holdings PLC (Boldon, UK) and Diasorin Inc. (Stillwater, MN, USA), respectively’. Similarly, in a 2013 study [5], the authors stated that ‘serum 1.25-hydroxy VD3 and 25-hydroxy VD3 levels were determined using RIA kits from Immunodiagnostic Systems Holdings plc (Boldon, UK) and Diasorin Inc. (Stillwater, MN), respectively’. Despite using the same analytical methods and assay kits in both studies, the authors reported the measured analytes as total vitamin D metabolites in one case and as vitamin D_3_ metabolites in the other. However, an examination of the manufacturers’ documentation for these assay kits, as well as evidence from the existing literature using these methods, indicates that these assays measure total vitamin D metabolites rather than vitamin D_3_ alone [6,7]. Indeed, immunoassays, including radioimmunoassays (RIAs), generally do not distinguish between 25(OH)D_2_ and 25(OH)D_3_ and therefore report the total 25(OH)D, whereas chromatographic methods such as liquid chromatography–tandem mass spectrometry allow for separate quantification of 25(OH)D_2_ and 25(OH)D_3_ [8].

There are several claims in the comment [1] that demand addressing. The 2011 study by Nagasaka et al. [4] has been referred to as a ‘small adult study’. The authors enrolled 34 patients with PKU aged 20–35 years. Given the Japanese population in 2010 (128 million inhabitants), the median age of the Japanese population (approximately 44 years), and the prevalence of PKU in Japan (1:125.000), it is easy to calculate that this study comprised approximately 1/5 of all Japanese patients in this age group (or all patients living at that time in two major Japanese cities—Tokyo and Osaka). It documents the visibility problem of rare diseases well. There are a small number of patients and few studies, yet the same high expectations and requirements still stand as for common diseases. The use of a food-frequency questionnaire may result in nutritional recall bias, but, in any case, it does influence the assessment of vitamin concentrations (vitamin D intake was not an outcome of interest). The claim that conclusions about folates are inappropriate is not justified. Folate levels were proven to be higher in patients with PKU than in healthy subjects, with and without excluding studies that could be the cause of potential bias [2]. The comment on the conclusion regarding 1,25(OH)D that overlooks limitations in metabolite selection and assay comparability also appears unjustified, as only one metabolite was measured across all eligible studies [4,5,9]. Merging different instruments used to assess vitamin D concentrations may introduce significant bias when comparing vitamin status between patients with PKU across studies. However, in the meta-analysis, we include both patients and healthy individuals who have been tested with the same method, thereby reducing/preventing bias.

In addition, Iqhrammullah [1] mentioned that changing the composition of protein substitutes over time introduces additional heterogeneity. This issue was addressed in the Discussion Section of our paper [2]. Moreover, Iqhrammullah [1] suggests that this was not reflected in the Abstract, although our Abstract clearly stated limitations related to heterogeneity and variability: ‘the main limitations of the evidence include a limited number of studies and their heterogeneity and variability in patients’ compliance’ [2]. Due to strict word count limitations, a more detailed discussion could not be included in the Abstract. It is worth noting that there are many other factors resulting in potential heterogeneity among the studies considered in our meta-analysis, including but not limited to the use of different biological samples (plasma or serum), changes in treatment strategies, compliance among PKU patients, the severity of PKU, patient demographics (age, sex, and ethnicity), as well as environmental factors, such as sunlight exposure and regional dietary patterns. All of them were mentioned in the paper as potential factors influencing the obtained results [2].

We agree with the author of the comment [1] in that the results of the meta-analysis [2] are more influenced by certain studies, particularly those by Nagasaka et al. [4,5]. We addressed this in our paper [2]. In addition to presenting the main results of the meta-analysis, we conducted additional analyses, excluding studies with a high risk of bias and a sensitivity analysis, to allow readers to assess the certainty of the evidence. As noted by the commenter, the Cook’s distance threshold was exceeded in the 2013 study by Nagasaka et al. [5]. However, it should be highlighted that this metric should not be used as a criterion for excluding a study from a meta-analysis [10,11]. Nevertheless, with no regard for which analysis (including all studies or a subset of studies) the results are considered, the major message for the PKU field remains unchanged: ‘…individuals with PKU can attain adequate vitamin status similar to that of their healthy counterparts’ [2].

Nagasaka et al. [4] speculated that one of the reasons for the higher 1,25(OH)_2_D levels reported in subjects with PKU compared to healthy individuals might indicate enhanced renal 1α-hydroxylase activity. However, unlike the author of the comment [1], we interpret this as a hypothesis rather than a conclusion, which requires confirmation in future research. As elucidating the reasons for these findings was not the aim of our meta-analysis, we emphasize in the Discussion that ‘potential high levels of 1,25-dihydroxyvitamin D in PKU patients are very interesting and may form the basis for future investigations’ [2].

Nevertheless, the results of our meta-analysis, although very important for PKU considerations, should be interpreted with caution, not only because of significant heterogeneity among the included studies but also because of the small number of studies in this systematic review. The limitations are mentioned above and in the published paper [2].

## Abbreviations

The following abbreviations are used in this manuscript:

PKU	Phenylketonuria
RIA	Radioimmunoassay
1,25(OH)_2_D	1,25-dihydroxyvitamin D
1,25(OH)_2_D_3_	1,25-dihydroxyvitamin D_3_
25(OH)D	25-hydroxyvitamin D
25(OH)D_2_	25-hydroxyvitamin D_2_
25(OH)D_3_	25-hydroxyvitamin D_3_
